# Differential diagnosis of thrombotic microangiopathy in nephrology

**DOI:** 10.1186/s12882-017-0727-y

**Published:** 2017-10-28

**Authors:** T. Sakari Jokiranta, Ondrej Viklicky, Saleh Al Shorafa, Rosanna Coppo, Christoph Gasteyger, Manuel Macia, Tatiana Pankratenko, Mohan Shenoy, Oğuz Soylemezoglu, Michel Tsimaratos, Jack Wetzels, Hermann Haller

**Affiliations:** 1 Research Programs Unit, University of Helsinki and Helsinki University Central Hospital, Helsinki, Finland; 2 Institute for Clinical and Experimental Medicine, Prague, Czech Republic; 3 Hospital Qatif Central Hospital, Qatif, Saudi Arabia; 4 Fondazione Ricerca Molinette, Regina Margherita Hospital, Turin, Italy; 5 Alexion Pharma GmbH, Zurich, Switzerland; 6 Hospital Virgen de la Candelaria, Santa Cruz de Tenerife, Spain; 7 M.F. Vladimirskiy Moscow Regional Research and Clinical Institute, Moscow, Russia; 8 Royal Manchester Children’s Hospital, Manchester, UK; 9 Gazi University Hospital, Ankara, Turkey; 10 Pédiatrie Multidisciplinaire-Hôpital de la Timone, Marseille, France; 11 Radboud University Medical Centre, Nijmegen, Netherlands; 12 Department of Nephrology and Hypertension, Hannover Medical School, Hannover, Germany

**Keywords:** aHUS, AKI, Complement, Haemolytic anaemia, Kidney diseases, Survey, Thrombocytopenia, TMA, TTP

## Abstract

**Background:**

The differential diagnosis of thrombotic microangiopathy (TMA) is complex however the rapid diagnosis of the underlying condition is vital to inform urgent treatment decisions. A survey was devised with the objective of understanding current practices across Europe and the Middle East, and of challenges when diagnosing the cause of TMA.

**Methods:**

Over 450 clinicians, from 16 countries were invited to complete an online survey.

**Results:**

Of 254 respondents, the majority were nephrologists, had >10 years’ experience in their specialty, and had diagnosed a patient with TMA. The triad of thrombocytopenia, haemolytic anaemia and acute kidney injury are the main diagnostic criteria used. Responses indicate that a differential diagnosis of TMA is usually made within 1–2 (53%) or 3–4 days (26%) of presentation. Similarly, therapy is usually initiated within the first 4 days (74%), however 13% report treatment initiation >1-week post-presentation. Extrarenal symptoms and a panoply of other conditions are considered when assessing the differential diagnosis of TMA. While 70 and 78% of respondents stated they always request complement protein levels and ADAMTS13 activity, respectively. Diagnostic considerations of paediatric and adult nephrologists varied. A greater proportion of paediatric than adult nephrologists consider extrarenal manifestations clinically related to a diagnosis of TMA; pulmonary (45% vs. 18%), gastrointestinal (67% vs. 50%), CNS (96% vs. 84%) and cardiovascular (54% vs. 42%), respectively. Variability in the availability of guidelines and extent of family history taken was also evident.

**Conclusions:**

This survey reveals the variability of current practices and the need for increased urgency among physicians in the differential diagnosis of TMA, despite their experience. Above all, the survey highlights the need for international clinical guidelines to provide systematically developed recommendations for understanding the relevance of complement protein levels, complement abnormalities and ADAMTS13 testing, in making a differential diagnosis of TMA. Such clinical guidelines would enable physicians to make a more rapid and informed diagnosis of TMA, therefore initiate effective treatment earlier, with a consequent improvement in patient outcomes.

**Electronic supplementary material:**

The online version of this article (doi: 10.1186/s12882-017-0727-y) contains supplementary material, which is available to authorized users.

## Background

Thrombotic microangiopathy (TMA) manifests as a histological lesion of the microvasculature characterised by thickened and swollen vessel walls, detachment of endothelial cells, build-up of proteins and cell lysis material in the sub-endothelial space, and obstruction of the vascular lumen by platelet thrombi [1].

Thrombotic thrombocytopenic purpura (TTP) and haemolytic uraemic syndrome (HUS) are the two most common clinical conditions characterised by TMA lesions but have differing aetiology, pathophysiology and management strategies [1]. TTP is a systemic disorder of microvascular thromboses due to a deficiency in a disintegrin and metalloproteinase with a thrombospondin type 1 motif, member 13 (ADAMTS13) activity, as a result of autoantibodies or genetic mutations. A severe reduction of ADAMTS13 activity (<10%) results in the formation of ultra-high molecular weight von Willebrand factor multimers, causing aggregation of platelets and end-organ ischaemia [2]. HUS meanwhile, is a clinical syndrome characterised by the obstruction of microvasculature (most commonly in the kidney) by platelet-fibrin thrombi despite normal ADAMTS13 activity. Shiga-toxin producing *Escherichia coli* (STEC) is the most common cause of HUS (STEC-HUS) [3]. History of an accompanying condition, including the presence of non-STEC infections such as *Streptococcus pneumoniae* or influenza virus infection, malignant hypertension, transplantation, pregnancy and child birth, or drug usage may suggest a diagnosis of secondary TMA [3]. The atypical form of HUS (aHUS) is a rare, life-threatening disease of chronic, uncontrolled complement activation that leads to TMA with severe organ damage. The differential diagnosis of aHUS requires the exclusion of TTP and STEC-HUS [1]. The rapid progression of TMA, associated with potentially irreversible damage to organs in patients with aHUS, indicates a need for urgent treatment. Historically, disease outcomes have been poor, despite previous practice to manage aHUS by intensive plasma exchanges [4]. More recently, eculizumab, a humanised monoclonal antibody that binds to the complement protein C5 preventing the formation of the membrane attack complex, has been shown to be effective in treating patients with aHUS [5–7]. A recent report demonstrated a greater and more sustained recovery in renal function when eculizumab therapy is initiated within 7 days of aHUS onset [8].

The differential diagnosis of TMA is complex but important to inform treatment decisions. Consensus guidelines for the differential diagnosis of TMA were updated in 2015 [1], however real-world evidence on the current diagnosis and treatment practices has not been reported. We hypothesised that there are areas of uncertainty in the work-up of patients with TMA, leading to potential delays in establishing the final diagnosis, putting patient’s lives at a higher risk.

We devised a survey with the objective of understanding current practices in the diagnosis of TMA and aHUS across Europe and the Middle East and challenges when diagnosing the cause of TMA.

## Methods

### Survey

The survey was developed by the authors in 2015. Initial questions were proposed in a draft version which was discussed by all the authors. The draft was subsequently revised, including the removal and addition of questions and possible responses. The final version of the survey was distributed to the authors for review as an additional quality control measure and was distributed between January and March 2016, using the online tool surveymonkey.com. The authors distributed the survey to paediatric and adult nephrology centres, multi-disciplinary hospitals, and academic clinics within their countries or regions. The survey was composed of 36 questions with multiple choice answers provided (Additional file 1: Table S1). Questions included characteristics of the respondent’s place of work, and their experience in the diagnosis and management of patients with TMA and aHUS. No personal information was collected or stored. The survey used adaptive questioning (certain questions were only displayed based on previous responses) to avoid redundant questions. Respondents could review and change their answers, through a “Back” button function. The survey was translated into Russian and German for non-English speaking participants in those regions. Participants were informed that data collected would be published in a research article.

### Participants

An email invitation with a link to the survey was sent to over 450 clinicians, from 16 countries, Belgium, Czech Republic, Denmark, Finland, France, Germany, Italy, Netherlands, Norway, Russia, Saudi Arabia, Spain, Sweden, Switzerland, Turkey and UK.

### Data analysis

The responses were compiled in a spreadsheet and descriptive analyses were performed. Participant and centre name were not requested in the survey. Not all respondents answered the survey in its entirety, therefore the number of responses per individual question are stated. Results are reported as counts and percentages of responses received.

## Results

### Participation

Two hundred and fifty-four clinicians from 16 countries participated: Belgium (2), Czech Republic (2), Denmark (2), Finland (17), France (24), Germany (14), Italy (21), Netherlands (47), Norway (4), Russia (42), Saudi Arabia (1), Spain (29), Sweden (3), Switzerland (1), Turkey (37) and UK (8). Eighty-three percent of the 254 respondents completed the survey in its entirety. Of the 254 respondents, 82% were nephrologists, 69% had more than 10 years’ experience in their specialty, and 89% had diagnosed a patient with TMA. Table 1 describes the characteristics of clinicians that participated in the survey. Additional professionals responding included haematologists, paediatricians and intensive care unit specialists.

**Table 1:** Respondent characteristics

Question and response options	*n* (%)
Institute type:	
Academic centre	216 (85)
Private hospital	35 (14)
Private practice	6 (2)
Department/division type:	
Nephrology	133 (52)
Paediatric nephrology	68 (27)
Intensive care	6 (2)
Emergency ward	0 (0)
What is your current speciality?	
Adult nephrologist	132 (52)
Paediatric nephrologist	75 (30)
ICU specialist	6 (2)
How many years have you been practicing your current speciality?	
< 1 year	4 (2)
1–5 years	29 (11)
6–10 years	47 (18)
> 10 years	175 (69)
Have you ever personally diagnosed a patient with TMA (or validated the diagnosis of TMA in a patient referred to you)?^a^	
Yes	221 (89)
No	28 (11)
Are there any guidelines in place for the diagnosis of TMA at your hospital?^b^	
Yes	121 (54)
No	105 (46)
Are the guidelines local to your institute, national or international?^c^	
Local	41 (34)
National	47 (39)
International	34 (28)

Percentages are based on 254 responses, with the exception of ^a^(*n* = 248), ^b^(*n* = 226) and ^c^(*n* = 122)

### Diagnosis of TMA

**Fig. 1:**
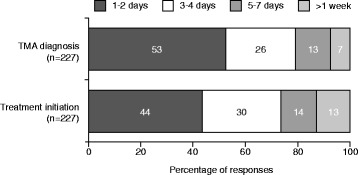
Time taken to make a differential diagnosis of TMA and initiate therapy. The aim was to determine the time taken to take specific action following differential diagnosis and not to define the treatments provided

**Fig. 2:**
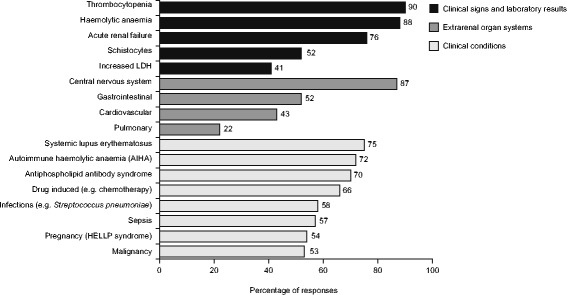
The main clinical signs and laboratory results (*n* = 229), extrarenal organ systems (*n* = 229) and clinical conditions (*n* = 227) considered when assessing the differential diagnosis of TMA

Results show that a differential diagnosis of TMA is usually made within 1–2 (53%) or 3–4 days (26%) of presentation. Similarly, therapy is usually initiated within 1–2 (44%) or 3–4 days (30%), however 13% reported treatment initiation >1-week post-presentation (Fig. 1). Respondents indicated that thrombocytopenia, haemolytic anaemia and acute kidney injury (AKI) are the main clinical diagnostic criteria used (Fig. 2). In working-up a TMA diagnosis 70 and 78% of respondents stated they always request complement protein levels and ADAMTS13 activity, respectively. However, only 66% agreed that an ADAMTS13 activity >10% rules out TTP. Neurological, gastrointestinal, cardiovascular and pulmonary symptoms and a variety of other conditions are considered when assessing the differential diagnosis (Fig. 2). Twenty-two percent of respondents reported that they complete a full family tree when they diagnose a patient with TMA. Over 65% of respondents indicated that renal biopsies are only requested when a diagnosis is not clear.

### Diagnosis of aHUS

**Fig. 3:**
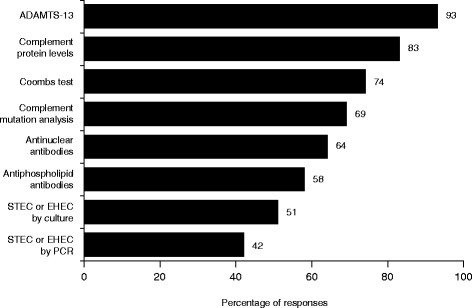
Tests requested to work up the diagnosis of aHUS (*n* = 221)

For the diagnosis of aHUS, 93% of respondents request ADAMTS13 activity tests and 83% request complement protein levels (Fig. 3). Fifty-four percent of responding clinicians stated that guidelines for the diagnosis of TMA are available within their hospital (Table 1). These guidelines were similarly described as being local to their institute, national or international. Approximately half of the respondents routinely perform genetic testing, and this is paid for by the hospital in 46% of cases.

### Paediatric and adult nephrologist diagnostic practices

A sub-analysis compared the current diagnostic practices of paediatric (*n* = 75) and adult nephrologists (*n* = 132; Table 2). A greater proportion of paediatric than adult nephrologists considered pulmonary, gastrointestinal, central nervous system (CNS) and cardiovascular manifestations clinically related to a diagnosis of TMA. As would be expected when assessing the differential diagnosis, paediatric nephrologists more often considered infections and cobalamin metabolism disorders than adult nephrologists, and less often considered scleroderma, malignancy and HELLP (haemolysis, elevated liver enzyme levels, and low platelet levels) syndrome. Paediatric nephrologists also requested tests for enterohaemorrhagic *Escherichia coli* (EHEC) infection more frequently than adult nephrologists.

**Table 2:** Comparison of paediatric and adult nephrologist’s diagnostic practices for TMA

	Response *n* (%)^a^	
	Paediatric nephrologist *n* = 75	Adult nephrologist *n* = 132
Apart from renal symptoms, which other organ manifestations would you consider clinically related to a diagnosis of TMA?		
Central nervous system	64 (96)	103 (84)
Gastrointestinal	45 (67)	62 (50)
Cardiovascular	36 (54)	52 (42)
Pulmonary	30 (45)	22 (18)
What other conditions do you usually consider when assessing the differential diagnosis of TMA?		
Antiphospholipid syndrome	41 (60)	96 (79)
Autoimmune haemolytic anaemia	42 (62)	96 (78)
Clotting disorder	45 (66)	25 (21)
Cobalamin metabolism disorder	18 (26)	43 (35)
Drug induced	44 (65)	87 (71)
HIV	15 (22)	38 (31)
Infections	55 (81)	58 (48)
Malignancy	22 (32)	78 (64)
Pregnancy (HELLP syndrome)	15 (22)	86 (71)
Scleroderma	9 (13)	48 (39)
Sepsis	36 (53)	71 (58)
Systemic lupus erythematosus	49 (72)	103 (84)
Other^b^	4 (7)	11 (9)
How extensive is your investigation of the patients’ family history?		
Ask the patient	21 (31)	75 (61)
Investigate immediate family	19 (28)	28 (23)
Complete a full family tree	28 (41)	19 (16)
Do you routinely perform genetic testing?		
Yes	46 (69)	49 (41)
No	21 (31)	70 (59)
Are there any guidelines in place for the diagnosis of TMA at your hospital?		
Yes	49 (72)	51 (42)
No	19 (28)	70 (58)
When the differential diagnosis of TMA was aHUS, what was the most challenging aspect of the diagnosis?^c^		
Absence of guidelines	2.1	2.5
Delay in getting some laboratory results	3.7	3.4
Absence of a single and reliable diagnostic test	3.6	3.5
Heterogeneity of disease presentation	2.9	3.5

^a^Percentage obtained from the number of responses received for individual questions

^b^Fifteen respondents also selected “other” considerations as free text; these included malignant hypertension (*n* = 6), viral infections (*n* = 3; H1N1, hantavirus and parvovirus), disseminated intravascular coagulation (*n* = 2), transplantation (*n* = 2), vasculitis (*n* = 2), aHUS (*n* = 1), HUS (*n* = 1), pre-eclampsia (*n* = 1), TTP (*n* = 1), and AKI of unknown aetiology (*n* = 1). Clinicians could include >1 answer in response to ‘Other’

^c^Clinicians were asked to grade their response from 1 (least challenging) to 5 (most challenging), data are presented as mean score. HELLP, haemolysis, elevated liver enzyme levels, and low platelet levels; HIV, human immune deficiency virus; TMA, thrombotic microangiopathy

Paediatric nephrologists are more likely to complete a full family tree when investigating the patients’ family history compared with adult nephrologists (41% vs 16%). Paediatric nephrologists are also more likely to routinely request genetic testing than adult nephrologists (69% vs 41%). Guidelines for the diagnosis of TMA are more readily available to paediatric nephrologists (72%) than adult nephrologists (42%).

The most challenging aspect of an aHUS diagnosis for paediatric nephrologists is the delay in getting some laboratory results. However, the absence of a single reliable diagnostic test and the heterogeneity of disease presentation are the most challenging aspects of diagnosing aHUS for adult nephrologists (Table 2).

## Discussion

This survey provides a large source of information describing current practices in the differential diagnosis of TMA amongst experienced clinicians in 16 countries in Europe and the Middle East.

Clinical guidelines are an important tool to facilitate clinical decision making and promote the delivery of evidence-based best practice [9]. However, clinical guidelines for rare diseases are often difficult to formulate due to a lack of quality evidence, surveys of clinical practices, and data about patients’ opinion (https://www.iqwig.de/download/V10-01_Executive_Summary_Evidence_for_guidelines_on_rare_diseases.pdf). Recommendations provided within current guidelines are opinions, based on the best available scientific evidence [1, 10–12].

Responses suggest the awareness, use, or perceived importance of published recommendations [1, 10–12] available for the management of patients with TMA is inconsistent between countries and even within hospitals from the same country. Adult nephrologists seem to be less aware of published guidelines, however this was rated as the least challenging aspect of making an aHUS diagnosis suggesting limited clinical impact.

In accordance with recent recommendations [1, 10–12] the triad of haemolytic anaemia, thrombocytopenia and AKI were the three main criteria indicating a diagnosis of TMA. However, the presence of schistocytes and increased lactate dehydrogenase (LDH) were not considered primary criteria in the diagnosis of TMA by many physicians. Considering the rapid progression and severity of TMA, guidelines recommend a rapid differential diagnosis to be established allowing for appropriate supportive measures to be taken with the first 48 h from admission [1]. In practice however, approximately half of clinicians state a diagnosis of TMA takes more than 3 days. Similarly, therapeutic strategies are initiated after 3 days or more in the majority of cases (57%). These data support those from a recent survey conducted by the aHUS Alliance whereby less than 55% of 233 patients with aHUS received their diagnosis within a week of presentation [13]. Determining ADAMTS13 activity levels and the absence of STEC infection in a timely manner (<24 h) is crucial in discriminating TTP from other TMA [1, 2]. By identifying ADAMTS13 activity levels early, appropriate treatment can be initiated quickly, and patients without TTP avoid unnecessary plasma exchange. Thereby patient outcomes may be improved.

Despite being part of recommended algorithms for the differential diagnosis of TMA [1, 10] only two-thirds of respondents agreed that an ADAMTS13 activity >10% rules out TTP. aHUS is a rare disease and as such awareness of the role of ADAMTS13 testing in the differential diagnosis may be low. One guideline recommends ADAMTS-13 activity <5–10% is indicative of TTP [1]. However, other reports have used <5% [11] and <10% [14] as a reference value to inform on diagnosis. It is possible that some centres may adopt ADAMTS-13 activity >5% as a lower limit to rule out a differential diagnosis of TTP, which may partially explain this result. Alternatively, there are also reports stating 10–25% of TTP patients have normal ADAMTS-13 activity [15] with activity levels varying from 33 to 100% among patients with apparent idiopathic TTP [16]. The response received in the survey may reflect that some physicians are not medically convinced that ADAMTS13 > 10% rules out TTP in 100% of cases.

The systemic nature of TMA leads to the involvement of the microvasculature of organs other than the kidney, with neurological manifestations being the most common [17]. This is reflected in 87% of respondents stating they consider neurological symptoms when considering a TMA diagnosis. However, it is important to consider that many other organs can be affected by TMA [18].

One guideline recommends a complete and detailed clinical history should be made for all TMA patients, including personal and family history [1], unless an EHEC infection is obvious. Current data suggest this is not routinely the case, as half of respondents reported that a family history would be determined by asking the patient only. A more thorough family history would be beneficial, as the majority of the reported aHUS associated mutations are dominant but with incomplete penetrance [17].

Gene mutations or polymorphisms affecting complement regulators or proteins are identified in 60–70% of aHUS patients [19, 20]. While identification of a complement mutation or anti-complement factor H (CFH) inhibitory antibody is not required to make a diagnosis of aHUS or initiate therapy, it does inform on prognosis and risk of recurrence [1, 8, 20, 21]. In this study, genetic testing is routinely performed by just under half of the clinicians managing patients with aHUS, but more commonly by paediatric nephrologists. In contrast, a survey conducted by the aHUS Alliance observed 84% of patients with aHUS have had or are awaiting to undergo genetic testing [13]. In our survey the percentage was clearly lower, in addition, a family tree was completed for only 41% of paediatric patients and 16% of adult patients. Our survey does not allow understanding of the reasons for this relatively low proportion for genetic testing. The lack of guidelines, the cost, the time taken to get results, as well as difficulty in interpreting the results, may influence the extent to which a family history is investigated. The introduction of uniform reporting, tailored to non-geneticists may increase confidence in physicians requesting genetic tests. This may in turn result in a more consistent application of genetics to inform on prognosis and the risk of recurrence, and provide information to expand our understanding on the natural course of aHUS.

There are limitations to interpreting data collected by surveys. Here, the survey was sent to email addresses available to the authors and may have led to some survey bias. Additionally, not all countries were equally represented, however despite this the survey covered a large and unprecedented amount of countries. Secondly, we cannot be sure of the precise number of recipients, as clinicians may have forwarded the survey to colleagues. However, we estimate a satisfactory response rate compared with the average for web-based surveys [22]. Based on a meta-analysis of 68 web-based surveys in 49 studies [23], we estimate 641 clinicians would have been invited for our response rate to be similar to the 39.6% reported [23]. Thirdly, the majority of responses were provided by experienced nephrologists and not physicians from other specialties. As such, our results may not be applicable to diagnostic practices of non-nephrology departments, where awareness of this ultra-rare disease is likely to be lower. Finally, the survey did not undergo a formal validation process; however, three drafts were subsequently developed and reviewed by the authors prior to circulating the final survey to a large audience.

We hypothesised that there were areas of uncertainty in the work-up of patients with TMA, leading to potential delays in establishing the final diagnosis, putting patient’s lives at a higher risk. This survey addressed many elements of the hypothesis however other aspects warrant further investigation. The current survey focused on the diagnosis, not on the management, of TMA. Real-world information on the management practices of clinicians treating patients with TMA would be of interest and a worthwhile topic for a follow-up survey in the future.

## Conclusion

This survey evaluated current clinical practices of experienced paediatric and adult nephrologists in the diagnosis of TMA including aHUS. We describe the variable practices in the differential diagnosis of TMA amongst respondents, despite their considerable clinical experience. Our survey suggests that some countries lack national guidelines and these should be put in place where possible. Guidelines would serve to raise awareness of the availability and relevance of ADAMTS13 and STEC testing to discriminate TTP and STEC-HUS from aHUS. These clinical guidelines could potentially increase awareness of the evidence for genetic testing after the initial therapy has been started and the importance of conducting a full family history, when presented with a genetic disease as a possible diagnosis. Our results also suggest that having guidelines does not guarantee their implementation; future activities should focus more on the dissemination of knowledge and education, to encourage guideline implementation. Continuing medical education courses on diagnostic and treatment practices for TMA may be of benefit to clinicians. Finally, there is the need for clear and practical guidelines for general practitioners and primary care doctors, in order to accelerate the diagnostic process. Systematically developed guidelines will aid clinicians in diagnosing and managing TMA, enabling a more rapid and informed diagnosis and initiation of effective treatment earlier, with a consequent improvement in patient outcomes.

## Additional file

Additional file 1: Table S1. Survey questions. (DOCX 16 kb)

## Abbreviations

ADAMTS13: A disintegrin and metalloproteinase with a thrombospondin type 1 motif, member 13
aHUS: Atypical haemolytic uraemic syndrome
AKI: Acute kidney injury
CFH: Complement factor H
CNS: Central nervous system
EHEC: Enterohaemorrhagic *Escherichia coli*
HELLP: Haemolysis, elevated liver enzyme levels, and low platelet levels
HIV: Human immune deficiency virus
HUS: Haemolytic uraemic syndrome
LDH: Lactate dehydrogenase
PCR: Polymerase chain reaction
STEC: Shiga toxin-producing *Escherichia coli*
TMA: Thrombotic microangiopathy
TTP: Thrombotic thrombocytopenic purpura
