# Supplementary material for: Differential diagnosis of thrombotic microangiopathy in nephrology
Source: BMC Nephrol. 2017 Oct 28;18:324. doi: 10.1186/s12882-017-0727-y (PMC5660444; doi:10.1186/s12882-017-0727-y)
Supplement: Additional file 1: Table S1. — Survey questions. (DOCX 16 kb) [file 12882_2017_727_MOESM1_ESM.docx]

**Table S1.** Survey questions

| **General information**   1. In what country do you work?   Drop down menu available   1. Institute type:   □ Private practice □ Private hospital □ Academic centre   1. Department/division type?   □ Nephrology □ Paediatric nephrology □ Intensive care □ Emergency ward □ Other, please specify   1. What is your current speciality?   □ Adult nephrologist □ Paediatric nephrologist □ ICU specialist □ Other, please specify   1. How many years have you been practicing your current speciality?   □ <1 year □ 1–5 years □ 6–10 years □ >10 years |
| --- |
| **Section 1. Your experience of diagnosing TMA**   1. Have you ever personally diagnosed a patient with TMA (or validated the diagnosis of TMA in a patient referred to you)?   □ Yes □ No   1. Of the patients you have diagnosed with TMA in the last 5 years, in approximately how many has the final diagnosis been:  0 1–5 6–10 11–20 20+ STEC-HUS □ □ □ □ □ aHUS □ □ □ □ □ TTP □ □ □ □ □ Other TMA □ □ □ □ □ |
| **Section 2. Process of work-up for patients presenting with potential TMA**   1. Which of the following signs would primarily lead you to suspect TMA? Please select up to 4 options   □ AKI □ Coagulation prolongation/dysregulation □ Coombs negative □ Fever □ Gastrointestinal signs and symptoms □ Haemolytic anaemia  □ Increased LDH □ Neurological signs and symptoms □ Pulmonary bleeding □ Schistocytes □ Thrombocytopenia □ Other   1. Apart from renal symptoms, which other organ manifestations would you consider clinically related to a diagnosis of TMA?   □ Cardiovascular □ Central nervous system □ Gastrointestinal □ Pulmonary □ Other   1. Do you perform a renal biopsy for the diagnosis of TMA?   □ Always □ Never □ Only if diagnosis is not clear   1. Do you biopsy any other organs to diagnose TMA? □ Yes □ No 2. If yes, which other organs? □ Heart □ Lung □ Skin □ Other 3. What other conditions do you usually consider when assessing the differential diagnosis of TMA? □ Antiphospholipid syndrome □ Autoimmune haemolytic anaemia □ Clotting disorder □ Cobalamin metabolism disorder □ Drug induced □ HIV □ Infections  □ Malignancy □ Pregnancy (HELLP syndrome) □ Scleroderma □ Sepsis □ Systemic lupus erythematosus □ Other 4. Which tests do you request to differentially diagnose the cause of TMA? 1 = always, 2 = usually, 3 = rarely, 4 = never □ ADAMTS13 □ Antinuclear antibodies □ Antiphospholipid antibodies □ Complement protein levels □ Complement mutation analysis □ Homocysteine levels 5. How extensive is your investigation of the patients' family history? □ Ask the patient □ Investigate immediate family □ Complete a full family tree 6. In your experience, how long does it take to establish a diagnosis of TMA? □ 1–2 days □ 3–4 days □ 5–7 days □ >1 week 7. On average, how much time elapses from presentation to the initiation of specific therapeutic strategies? □ 1–2 days □ 3–4 days □ 5–7 days □ >1 week 8. Prior to confirmation of TMA diagnosis, what other specialists have been generally consulted for the signs and symptoms of the patient? □ Cardiologist □ Gastroenterologist □ Haematologist □ ICU specialist □ Infectious disease specialist □ Internal medicine specialist □ Nephrologist  □ Neurologist □ Obstetrician □ Paediatrician □ Paediatric nephrologist □ Surgeon □ Other 9. Are there any guidelines in place for the diagnosis of TMA at your hospital? □ Yes □ No 10. Are the guidelines: □ Local to your institute □ National □ International |
| **Section 3. Diagnosis of aHUS and subsequent management**   1. Have you ever personally diagnosed aHUS at your hospital? □ Yes □ No 2. How many aHUS patients have been referred to your institution within the last 5 years? □ 0 □ 1 □ 2–5 □ 6–10 □ >10 3. How many patients with aHUS are you currently managing? □ 0 □ 1 □ 2–5 □ 6–10 □ >10 4. What tests do you request to work-up the diagnosis of aHUS? □ ADAMTS13 □ Antinuclear antibodies □ Antiphospholipid antibodies □ Complement mutation analysis □ Complement protein levels  □ Coombs test □ STEC or EHEC □ STEC or EHEC by culture □ STEC or EHEC by PCR 5. Do you request ADAMTS-13 testing? □ Yes □ No 6. Which ADAMTS-13 tests do you have available? □ ADAMTS-13 activity □ ADAMTS-13 antibodies 7. Why not? □ Not available □ Don’t know □ Other 8. At what point in the patient diagnosis/treatment process do you draw blood for ADAMTS-13 testing? □ Prior to plasmapheresis □ After plasmapheresis □ Either to or after plasmapheresis 9. Approximately how many days does it take to get your ADAMTS-13 test result? □ 1 day or less □ 2–3 days □ 4–7 days □ >1 week 10. Do you routinely perform genetic testing? □ Yes □ No 11. Who pays for genetic testing in your hospital/region? □ Patient (without reimbursement) □ Government □ Hospital □ Insurance company □ Other 12. How quickly do you get results from genetic testing? □ <1 week □ 1–4 weeks □ >4 weeks but <3 months □ >3 months 13. Which genes are tested? □ ADAMTS-13 □ Coagulation or fibrinolysis proteins (e.g. plasminogen) □ CFHR-proteins □ Complement C3 □ Complement gene rearrangements and/or deletions □ Complement proteins but I do not know which □ Factor B □ Factor D □ Factor H □ Factor I □ MCP □ Other 14. Do you request tests for CFH (factor H) auto-antibodies? □ Yes □ No 15. Which is the most challenging aspect of aHUS diagnosis? 1 = least challenging, 5 = most challenging □ Absence of guidelines □ Delay in getting some laboratory results □ Absence of a single and reliable diagnostic test □ Heterogeneity of disease presentation 16. To what extent do you agree with the following statements related to the diagnosis of aHUS? 1 = disagree entirely, 5 = agree entirely □ ADAMTS-13 activity >10% rules out a diagnosis of severe ADAMTS-13 deficiency (TTP) □ A clinical diagnosis of aHUS could be considered in patients presenting with a complement-amplifying condition and TMA which does not improve after removal of the condition □ If performed for diagnosis, results from genetic tests are received quickly enough □ Identification of a genetic complement mutation is not required for aHUS diagnosis |

ADAMTS13, a disintegrin and metalloproteinase with a thrombospondin type 1 motif, member 13; aHUS, atypical haemolytic uraemic syndrome; CFH, complement factor H; CNS, central nervous system; EHEC, enterohaemorrhagic Escherichia coli; HELLP, haemolysis, elevated liver enzyme levels, and low platelet levels; HIV, human immune deficiency virus; HUS, haemolytic uraemic syndrome; LDH, lactate dehydrogenase; PCR, polymerase chain reaction; STEC, Shiga toxin-producing Escherichia coli; TMA, Thrombotic microangiopathy; TTP, Thrombotic thrombocytopenic purpura.
